# Fluorescent Immunochromatography for Rapid and Sensitive Typing of Seasonal Influenza Viruses

**DOI:** 10.1371/journal.pone.0116715

**Published:** 2015-02-04

**Authors:** Akira Sakurai, Katsuyoshi Takayama, Namiko Nomura, Naoki Kajiwara, Masatoshi Okamatsu, Naoki Yamamoto, Tsuruki Tamura, Jitsuho Yamada, Masako Hashimoto, Yoshihiro Sakoda, Yoshihiko Suda, Yukuharu Kobayashi, Hiroshi Kida, Futoshi Shibasaki

**Affiliations:** 1 Department of Molecular Medical Research, Tokyo Metropolitan Institute of Medical Science, 2–1–6, Kamikitazawa, Setagaya-ku, Tokyo, 156–8506, Japan; 2 ADTEC Co., LTD., 1693–6 Yokkaichi, Usa-City, Oita, 879–0471, Japan; 3 Laboratory of Microbiology, Department of Disease Control, Graduate School of Veterinary Medicine, Hokkaido University, Sapporo, 060–0818, Japan; 4 Department of Microbiology and Cell Biology, Tokyo Metropolitan Institute of Medical Science, 2–1–6, Kamikitazawa, Setagaya-ku, Tokyo, 156–8506, Japan; 5 Konica Minolta, Inc., No.1 sakura-machi, Hino-shi, Tokyo, 191–8511, Japan; 6 Global Station for Zoonosis Control, Global Institution for Collaborative Research and Education (GI-CoRE), Hokkaido University, Sapporo, 001–0020, Japan; University of Hong Kong, HONG KONG

## Abstract

Lateral flow tests also known as Immunochromatography (IC) is an antigen-detection method conducted on a nitrocellulose membrane that can be completed in less than 20 min. IC has been used as an important rapid test for clinical diagnosis and surveillance of influenza viruses, but the IC sensitivity is relatively low (approximately 60%) and the limit of detection (LOD) is as low as 10³ pfu per reaction. Recently, we reported an improved IC assay using antibodies conjugated with fluorescent beads (fluorescent immunochromatography; FLIC) for subtyping H5 influenza viruses (FLIC-H5). Although the FLIC strip must be scanned using a fluorescent reader, the sensitivity (LOD) is significantly improved over that of conventional IC methods. In addition, the antibodies which are specific against the subtypes of influenza viruses cannot be available for the detection of other subtypes when the major antigenicity will be changed. In this study, we established the use of FLIC to type seasonal influenza A and B viruses (FLIC-AB). This method has improved sensitivity to 100-fold higher than that of conventional IC methods when we used several strains of influenza viruses. In addition, FLIC-AB demonstrated the ability to detect influenza type A and influenza type B viruses from clinical samples with high sensitivity and specificity (Type A: sensitivity 98.7% (74/75), specificity 100% (54/54), Type B: sensitivity 100% (90/90), specificity 98.2% (54/55) in nasal swab samples) in comparison to the results of qRT-PCR. And furthermore, FLIC-AB performs better in the detection of early stage infection (under 13h) than other conventional IC methods. Our results provide new strategies to prevent the early-stage transmission of influenza viruses in humans during both seasonal outbreaks and pandemics.

## Introduction

Influenza, commonly known as "the flu", is an infectious disease of birds and mammals caused by negative-strand RNA viruses of the family *Orthomyxoviridae*, mainly Influenza type A virus (IAV), Influenza B type virus (IBV), and Influenza C type virus [1]. Seasonal outbreaks of IAVs and IBVs still cause considerably high morbidity, mortality, and global economic losses. IAVs are classified into 18 hemagglutinin (HA) and 11 neuraminidase (NA) subtypes (http://www.cdc.gov/flu/about/viruses/types.htm). The influenza viruses with many combinations of subtypes of these 2 surface proteins have been isolated from aquatic birds, poultry, and other avian species. A limited number of subtypes have been detected in humans. IAVs have the potential to become pandemic because of reassortment between avian and human viruses [2]. In contrast to IAVs, IBVs are only isolated from humans and seals, and consist of only 2 phylogenetic and antigenic lineages in human: the B/Victoria/2/87-like (Victoria) lineage and the B/Yamagata/16/88-like (Yamagata) lineage [3–5].

A recent pandemic of A/H1N1in 2009 (H1N1pdm) occurred upon reassortment between 2 swine influenza viruses, followed by triple-reassortment between the swine influenza virus and Eurasian-lineage swine influenza virus [6]. H1N1pdm caused more than 18,849 deaths in more than 214 countries [7]. The World Health Organization (WHO) announced on September 10, 2010 that the pandemic had transitioned into a post-pandemic phase [8]. The pathogenicity of H1N1pdm was initially estimated to be lower than that of the 1918 pandemic influenza virus “Spanish influenza”, but comparable to that of the 1957 pandemic influenza virus “Asian influenza” [9,10]. Subsequently, 2 separate estimates indicated that the case fatality ratio (CFR) of H1N1pdm was relatively similar to that of seasonal influenza in an average year [11,12], respectively. In Japan, there were only 85 confirmed deaths among estimated 12.6 million patients [13]. The low CFR in Japan was predicted to depend on rapid diagnosis and early treatment with neuraminidase inhibitors, such as oseltamivir and zanamivir [13]. These outcomes suggest that rapid diagnosis systems play an important role in clinical care for infected patients.

Recently, “variant” swine influenza viruses were isolated from humans [14]. An outbreak of influenza A H3N2 variant virus (H3N2v) occurred in the United States (12 states) in 2011 and 2012. This outbreak resulted in 309 human cases, including 16 hospitalizations and one death [15]. H3N2v infection has been associated with exposure to swine at agricultural fairs. H3N2v is characterized by the matrix segment of the viral gene from H1N1pdm [16]. The first H3N2v outbreak was reported in June 26, 2013 [17,18]. These reports indicated that variant swine influenza viruses have the potential to spread in humans.

Pandemic expansion of IAVs can be associated with a high mortality rate when the virus has been exposed to critical mutations, new combinations of surface antigens, or new reassortments. The outbreak of highly pathogenic avian influenza (HPAI) H5N1 virus in Hong Kong in 1997 was the first documented case of lethal infection [19–21] of humans with direct transmission from an avian species. Subsequently, HPAI H5N1 viruses have been spread by infected poultry in Asia, Europe, and Africa [22,23]. The WHO reported that the HPAI H5N1 virus has infected 620 individuals, causing 367 deaths (~59% mortality) as of February 15, 2013 [24].

A novel H7N9 influenza virus has caused 135 human infections and 44 deaths in China since February 18, 2013 [25–27]. Despite its high pathogenicity in humans, the H7N9 virus has low pathogenicity in avian hosts [28,29] and mice [30–32], and is likely to spread in birds or other animals without symptoms.

Taken together, these reports indicated the possibility of the occurrence of a pandemic caused by new animal influenza viruses, such as HPAI H5N1 virus, H3N2v, and H7N9 virus. If mutation of these viruses were to confer the ability for efficient human-to-human transmission, it might pose a serious threat to human health and the global economy.

Immunochromatography (IC) is an important rapid test for clinical diagnosis and surveillance of influenza viruses and can be completed in less than 20 min [33–35]. IC has, however, relatively low sensitivity (approximately 60%) [36]. The LOD has been improved, but not enough for the diagnosis and typing of influenza viruses in early phase of the infection. We previously reported an improved IC assay using antibodies conjugated with fluorescent beads (fluorescent immunochromatography; FLIC) for subtyping H5 IAVs (FLIC-H5) [37]. The LOD is significantly improved (50-fold higher) in combination with a newly-developed fluorescent reader [37].

Here, we modified the FLIC to be specific for typing IAVs and IBVs (FLIC-AB). The FLIC-AB can detect 16 laboratory strains of IAVs and 4 strains of IBVs. In clinical trials, FLIC-AB showed a significantly higher sensitivity than conventional ICs did in the diagnosis of over 200 influenza viruses isolated from patients in the two seasons of 2011 and 2012. The sensitivity was especially improved for the diagnosis of infected patients within 6h of the onset. Our results indicated that the FLIC-AB functions as a powerful tool for the diagnosis and typing of seasonal influenza viruses in the early stages of onset. This method would be particularly useful during pandemics and outbreaks.

## Results

### Establishment of FLIC for typing seasonal influenza viruses

Recently, the two commercialized IC kits, (1) Prorast-Flu (Mitsubishi Chemical Medience Corporation, Tokyo, Japan, http://www.medience.co.jp/english/index.html; **Prorast**) which uses a colloidal gold conjugated antibody, and (2) QuickNavi-Flu (Otsuka Pharmaceutical Co., Ltd; Tokyo, Japan, http://www.otsuka.co.jp/en/; **Quick**) which uses colored latex conjugated antibody, were widely used in many hospitals and clinics in Japan, and designated for typing seasonal IAVs and IBVs as control ICs. To focus on the development of a new IC kit with higher sensitivity than that of the commercialized kits, we established FLIC-AB by replacement of colloidal gold with fluorescent beads as previously described [37].

To evaluate the ability of FLIC-AB to detect 7 subtypes of influenza A viruses; A/WSN/1933 for H1N1; A/Hyogo/YS/2011 for H1N1 2009 pdm; A/swine/Missouri/2124514/2006 for H2N3; A/Aichi/2/1968 for H3N2; A/Indiana/08/2011 for H3N2v, and A/duck/Hokkaido/Vac-3/2007 for H5N1, A/Anhui/1/2013 for H7N9, and 2 subtypes of influenza B viruses; B/Tokyo/15480/2008 and B/Mass/3/1966 were tested in each single assay by using FLIC-AB or other control ICs; Prorast and Quick (Table 1). As briefly describe the calculations and methods used here, the sensitivity of FLICs was calculated using the exponential approximation of viral titer (pfu/mL) and the rate of signal/background (S/B). The cut-off S/B value was set at 1. The amount of viral titer at the intersection point of the exponential approximation and S/B = 1 was defined as the limit of detection (LOD) [37]. The results showed that FLIC-AB was able to detect 3 IAVs (H1N1, H1N1pdm, H3N2), and one IBV (B/Tokyo/15480/2008) with LOD of single digits in terms of pfu per reaction within 10 min and 15 min reading time of the device (Table 1). The LOD of FLIC-AB for 5 viruses (H1N1, H3N2, H3N2v, and H5N1, and 2 strains of IBVs) were more than 100 fold higher than those of control kits with Prorast and Quick. The LODs of H1N1pdm and H3N2v were 10 fold higher than those of the two control kits. H2N3 and H7N9 were only checked by FLIC-AB.

**Table 1:** LODs* of FLIC for typing influenza A viruses.

Type	Subtype or Lineage	Strain Name	LODs (pfu/reaction)			
			FLIC-AB 10 min	FLIC-AB 15 min	Prorast	Quick
A	H1N1	A/WSN/1933	9.3	6.1	10^3^	10^3^
A	H1N1 2009pdm	A/Hyogo/YS/2011	1.3	1.1	10^1^	10^1^
A	H2N3	A/swine/Missouri/2124514/2006	36.5	30	-**	-
A	H3N2	A/Aichi/2/1968	4.1	1.3	10^3^	10^3^
A	H3N2v	A/Indiana/08/2011	121	94.6	10^3^	10^3^
A	H5N1	A/duck/Hokkaido/Vac-3/2007	83.7	56.5	10^4^	10^3^
A	H7N9	A/Anhui/1/2013	595	628	-	-
B	Victoria	B/Tokyo/15480/2008	4.1	2.5	10^3^	10^3^
B	Laboratory strain***	B/Mass/3/1966	22.4	16.8	10^4^	10^4^

*LODs: Limit of Detections.

**not done.

***not classified.

To confirm the ability of FLIC-AB kits to detect multiple subtypes, 13 subtypes of avian IAVs and 2 lineages of IBVs were tested in each assay by FLIC-AB and the control kits (Prorast and Quick). Samples were applied for 15 min with fluorescent reader at the indicated dilution of viruses with each HA value as shown in Table 2. All subtypes of IAVs and both lineages of IBVs were detectable by FLIC-AB with at least 10-fold higher LOD than Prorast or Quick. Thus, FLIC-AB retains broad reactivity for IAVs and IBVs, with significantly higher LOD than 2 conventional IC methods. In addition, 5 sub-strains of HPAI H5N1 (Clade 0, 1.1, 2.3.2, and 2.3.4) [38] and H7N7 viruses were tested by FLIC-AB (Table 3). All HPAI viruses were detectable by FLIC-AB. Therefore, FLIC-AB can be used for surveillance of HPAI viruses at the first step. To confirm the specificity of FLIC-AB against influenza viruses, twenty-five common respiratory pathogens were applied to FLIC-AB kit. The titers of all bacteria and fungi used here were over 10^7^ colony-forming units/mL, and those of viruses were over 10^7^ TCID_50_/ml. These results confirmed that FLIC-AB has no cross-reactions with other pathogens used here (S1 Table). Thus, FLIC-AB does not produce negative background signals that can decrease specificity.

**Table 2:** Reactivities of FLIC for low pathogenic influenza A and B viruses.

Type	Subtype	Strain Name	HA value	Detected dilution rate		
				FLIC-AB	Prorast	Quick
A	H1N1	A/duck/Tottori/723/80	256	10^–5^	10^–4^	10^–5^
A	H3N8	A/duck/Mongolia/4/03	256	10^–6^	10^–4^	10^–4^
A	H4N6	A/duck/Czech/56	512	10^–5^	10^–4^	10^–5^
A	H5N2	A/duck/Pennsylvania/10218/84	128	10^–5^	10^–4^	10^–5^
A	H6N2	A/turkey/Massachusetts/3740/65	256	10^–5^	10^–4^	10^–4^
A	H7N7	A/seal/Massachusetts/1/80	512	10^–5^	10^–4^	10^–4^
A	H8N4	A/turkey/Ontario/6118/68	128	10^–4^	10^–3^	10^–4^
A	H9N2	A/turkey/Wisconsin/66	512	10^–5^	10^–4^	10^–5^
A	H10N7	A/chicken/Germany/N/49	2048	10^–5^	10^–4^	10^–5^
A	H12N5	A/duck/Alberta/60/76	512	10^–5^	10^–4^	10^–5^
A	H13N6	A/gull/Maryland/704/77	512	10^–5^	10^–4^	10^–5^
A	H14N5	A/mallard/Astrakhan/263/82	1024	10^–5^	10^–4^	10^–4^
A	H16N3	A/black-headed gull/Sweden/5/99	64	10^–4^	10^–3^	10^–4^
B	Victoria	B/Sendai/105/2007	128	10^–4^	10^–2^	10^–3^
B	Yamagata	B/Hokkaido/FO/2012	128	10^–4^	10^–2^	10^–3^

**Table 3:** Reactivity of FLIC-AB with H5 and H7 subtypes of influenza A viruses.

Subtypes	Strain Name	Reactivity	Clade
H5N1	A/Hong Kong/483/1997	+	0
H5N1	A/muscovy duck/Vietnam/OIE-559/2011	+	1.1
H5N1	A/whooper swan/Hokkaido/1/2008	+	2.3.2
H5N1	A/whooper swan/Hokkaido/4/2011	+	2.3.2
H5N1	A/peregrine falcon/Hong Kong/810/2009	+	2.3.4
H7N7	A/chicken/Netherlands/2586/2003	+	

Taken together, these results indicate that FLIC-AB may be a powerful tool for rapid typing of seasonal influenza A and B viruses, with high sensitivity and specificity.

### Sampling and clinical data

Nasal swab samples were taken from patients with a median age of 6-years old [ranging from 0- to 77-years old; quartile deviation of 3 to 11-years old]. Nasopharyngeal aspirates were taken from patients with a median age of 5-years old [ranging from 0- to 45-years old; quartile deviation of 2 to 8-years old]. Nasal discharge samples were taken from patients with a median age of 9-years old [ranging from 2 to 77-years old; quartile deviation of 6 to 12-years old]. 49.1% of the individuals studied were males, and some patients had vaccination against Influenza viruses. Samples were collected from Hokkaido, Tokyo, Oita, and other prefectures in the winter season of 2011 and 2012. Of the patients, the type of influenza type A or type B was determined when the control IC assays (Prorast and Quick) was positive. In addition, culture tests and quantitative real-time PCR (qRT-PCR) were performed to confirm diagnosis. Patients with a negative result in the control IC kit were diagnosed with the common cold, bronchitis, tonsillitis, pharyngitis, otitis media, or other acute respiratory disease.

### Identification of clinical samples

To analyze the sensitivity and specificity of FLIC-AB, nasal swabs, self-blow nasal discharge specimens, and nasopharyngeal aspirates from over 200 patients with suspected respiratory disease were tested by FLIC-AB, Prorast, Quick, or qRT-PCR using CDC protocols [39]. All samples were tested three times with each method. The raw data are shown in S2 Table for FLIC-AB vs. qRT-PCR, S3 Table for FLIC-AB and Prorast; S4 Table for FLIC-AB and Quick, respectively. The results of the sensitivity and specificity to compare FLIC-AB with qRT-PCR are summarized for typing influenza viruses from three different samples; nasal swab samples, nasal discharge specimens, and nasopharyngeal aspirates in Table 4, and those comparing FLIC-AB with Prorast in Table 5, and FLIC-AB with Quick in Table 6.

**Table 4:** Summary of sensitivity and specificity of FLIC-AB vs. qRT-PCR for typing influenza viruses from clinical samples.

	Target virus type	Samples	qRT-PCR	
			Sensitivity	Specificity
**Nasal swab samples**				
**FLIC-AB**	Influenza A	129	98.7% (74/75)	100% (54/54)
	Influenza B	145	100% (90/90)	98.2% (54/55)
**Self-blow nasal discharge specimens**				
**FLIC-AB**	Influenza A	125	94.6% (70/74)	100% (51/51)
	Influenza B	121	91.4% (64/70)	100% (51/51)
**Nasopharyngeal aspirates**				
**FLIC-AB**	Influenza A	142	98.6% (73/74)	97.1% (66/68)
	Influenza B	124	96.6% (56/58)	100% (66/66)

**Table 5:** Summary of sensitivity and specificity of FLIC-AB vs. Prorast for typing influenza viruses from clinical samples at symptom onset.

	Target virus type	Samples	Prorast	
			Sensitivity	Specificity
Nasal swab samples				
**FLIC-AB**	Influenza A	129	100% (73/73)	98.0% (55/56)
	Influenza B	146	100% (86/86)	91.7% (55/60)
Self-blow nasal discharge specimens				
**FLIC-AB**	Influenza A	131	100% (67/67)	95.3% (61/64)
	Influenza B	125	100% (61/61)	95.3% (61/64)
Nasopharyngeal aspirates				
**FLIC-AB**	Influenza A	144	100% (72/72)	95.8% (69/72)
	Influenza B	125	100% (55/55)	98.3% (69/70)

**Table 6:** Summary of sensitivity and specificity of FLIC-AB vs. Quick for typing influenza viruses from clinical samples at symptom onset.

	Target virus type	Samples	Quick	
			Sensitivity	Specificity
Nasal swab samples				
**FLIC-AB**	Influenza A	129	100% (74/74)	100% (55/55)
	Influenza B	151	100% (94/94)	91.2% (52/57)
Self-blow nasal discharge specimens				
**FLIC-AB**	Influenza A	131	100% (63/63)	89.7% (61/68)
	Influenza B	125	100% (60/60)	93.9% (61/65)
Nasopharyngeal aspirates				
**FLIC-AB**	Influenza A	144	100% (73/73)	97.2% (69/71)
	Influenza B	125	100% (55/55)	98.6% (69/70)

In clinical samples, FLIC-AB, and Prorast and Quick as control ICs, exhibited a high rate of positive and negative agreement in IAV and IBV detection. In addition, there were no FLIC-AB-negative/Prorast or Quick-positive results in our trials. All FLIC-AB-positive/Prorast or Quick-negative samples gave positive results by qRT-PCR, indicating that FLIC-AB has a higher sensitivity than other 2 classical ICs.

A summary of the sensitivity and specificity of FLIC compared to those of qRT-PCR are shown in Table 4. In nasal swab and nasopharyngeal aspirates cases, both the sensitivity and specificity of FLIC for typing influenza viruses were extremely high (96.6 to 100%). Although the specificity of FLIC in self-blow nasal discharge specimen cases remained high (100%), the sensitivity was lower than that in other sample types (IAV: 94.6%, IBV 91.4%). Nevertheless, the sensitivities for all sample types were high.

To analyze the performance of FLIC-AB in each clinical stage of infection, these results were re-analyzed with stratification according to time from the onset of symptoms (Table 7). In nasopharyngeal aspirates, the performance of FLIC-AB was equal to, or greater than those of Prorast and Quick. For the detection of IBVs from nasal swab samples less than 13 h of the onset of symptoms, there was a robust difference between the sensitivity of FLIC-AB and those of Prorast and Quick. Although FLIC-AB detected IBVs from nasal swabs with 100% (34/34) sensitivity at this earliest stage, Prorast or Quick did so with lower sensitivity (94.1% (32/34)) or (94.1% (32/34)), respectively. FLIC-AB was more sensitive than Prorast and Quick for self-blow nasal discharge specimens. The total sensitivities of FLIC-AB (IAVs: 97.3% (72/74), IBVs: 92.8% (65/70)) were higher than those of Prorast (IAVs: 93.2% (69/74), IBVs 79.7% (59/70)) and Quick (IAVs: 90.5% (67/74), IBVs; 77.0% (57/70)). In less than 13 h of onset, the sensitivities of IBVs varied considerably between these IC assays (FLIC-AB: 82.3% (36/39), Prorast: 79.5% (31/39), Quick; 76.9% (30/39)). These results indicated that FLIC-AB has an advantage in sensitivity over Prorast and Quick in early stage detection and typing of influenza infection.

**Table 7:** Sensitivity of FLIC-AB and control ICs for typing influenza viruses from clinical samples at symptom onset.

Hours from onset	Influenza A			Influenza B		
	FLIC-AB	Prorast	Quick	FLIC-AB	Prorast	Quick
**Nasal swab samples**						
<13h	97.4% (38/39)	97.4% (38/39)	97.4% (38/39)	100% (34/34)	94.1% (32/34)	94.1% (32/34)
13 to 24h	100% (31/31)	100% (31/31)	100% (31/31)	100% (23/23)	95.7% (22/23)	91.3% (21/23)
>24h	100% (3/3)	66.7% (2/3)	100% (3/3)	100% (12/12)	91.7% (11/12)	91.7% (11/12)
Unknown	100% (2/2)	100% (2/2)	100% (2/2)	100% (21/21)	100% (21/21)	100% (21/21)
Total	98.7% (74/75)	98.7% (74/75)	98.7% (74/75)	100% (90/90)	95.6% (86/90)	94.4% (85/90)
95% confidence interval	96.1 to 100%	50.8 to 100%	96.1 to 100%	100%	90.7 to 100%	87.4 to 100%
**Self-blow nasal discharge specimens**						
<13h	96.8% (30/31)	90.3% (28/31)	87.1% (27/31)	82.3% (36/39)	79.5% (31/39)	76.9% (30/39)
13 to 24h	94.4% (17/18)	94.4% (17/18)	94.4% (17/18)	90.0% (9/10)	80.0% (8/10)	70.0% (7/10)
>24h	100% (19/19)	94.7% (18/19)	89.5% (17/19)	90.9% (10/11)	90.9% (10/11)	90.9% (10/11)
Unknown	100% (6/6)	100% (6/6)	100% (6/6)	100% (10/10)	100% (10/10)	100% (10/10)
Total	97.3% (72/74)	93.2% (69/74)	90.5% (67/74)	92.8% (65/70)	79.7% (59/70)	77.0% (57/70)
95% confidence interval	93.1 to 100%	89.0 to 93.2%	85.8 to 100%	78.4 to 100%	75.6 to 100%	66.1 to 100%
**Nasopharyngeal aspirates**						
<13h	96.9% (31/32)	96.9% (31/32)	96.9% (31/32)	88.2% (15/17)	88.2% (15/17)	88.2% (15/17)
13 to 24h	100% (11/11)	100% (11/11)	100% (11/11)	100% (6/6)	100% (6/6)	100% (6/6)
>24h	100% (31/31)	96.8% (30/31)	100% (31/31)	100% (35/35)	97.1% (34/35)	97.1% (34/35)
Unknown	-	-		-	-	
Total	98.7% (73/74)	97.3% (72/74)	98.7% (73/74)	96.6% (56/58)	94.8% (55/58)	94.8% (55/58)
95% confidence interval	95.6 to 100%	95.6 to 100%	95.6 to 100%	84.3 to 100%	84.3 to 100%	84.3 to 100%

## Discussion

Clinical diagnostic tests for influenza viruses in outpatient departments or clinics are typically based on IC detection of influenza virus antigens [40]. The LOD of ICs is lower than that of PCR-based diagnostic methods [33]. To improve upon this shortcoming, we recently reported the FLIC system, or fluorescent IC, for subtyping HPAI H5N1 viruses [37]. Our data indicated that FLIC is one of the most sensitive diagnostic IC methods. In this study, we report the additional application of the FLIC method for clinical typing of seasonal influenza A and B viruses. LODs of FLIC-AB were shown to be in the single digit range in terms of pfu per reaction within 10 min. Although these LODs were lower than that of qRT-PCR (10^0^ to 10^–1^ pfu/reaction) [39], the sensitivities and specificities of FLIC-AB approached 100% in this study. Our results indicate that FLIC-AB test is sensitive enough to be suitable for early diagnosis of IAVs and IBVs in clinics and hospitals.

We previously reported that the LODs of FLICs for HPAI H5N1 viruses are 10–50 fold higher than those of colloidal gold ICs [37]. We now show that the sensitivities of FLIC-AB for typing IAVs and IBVs are more than 100 fold higher than those of the conventional Prorast and Quick kits for almost all viral strains tested in this study (Table 1). The difference in sensitivity between FLIC for HPAI H5N1 [37] and FLIC-AB may depend on the target antigens. In the case of the detection of HPAI H5N1 with FLIC, the HA antigen is sandwiched between 2 different HA specific antibodies. While in FLIC-AB, multimerized NP antigen complex including HA proteins is captured by anti-HA antibody and detected by NP specific antibody. The signals are amplified with NP detection because the ratio of NP after HA in the protein complex is more than 10 to 1. Therefore, the LODs of FLIC-AB are much higher than those of FLIC methods for HPAI H5N1. Fluorescent dye (630 nm) conjugated to detection antibodies can improve the LOD compared to that of conventional gold colloid conjugation. The LOD of FLIC-AB is one of the highest among IC methods, and is comparable to that of the silver amplification method [41]. Thus, FLIC-AB is expected to provide new strategies for rapid diagnosis of influenza viruses with high sensitivity.

Our clinical data indicated that FLIC-AB has an advantage in sensitivity even under poor sample conditions. Differences in sensitivity among the 3 IC methods were similar when using nasopharyngeal aspirates (Table 7). In contrast, FLIC-AB could detect IAVs and IBVs with higher sensitivities than Prorast and Quick when using self-blow nasal discharge specimens (Table 7). In addition, the sensitivities of IBV detection varied considerably among these ICs at the early detection stage. These data also indicate that FLIC-AB can detect a smaller amount of virus than conventional IC methods using clinical samples. Nasopharyngeal aspiration is the best way to collect viruses from patients, but collection by this method is more difficult for children than it is for nasal swab and self-blow nasal discharge specimens. It is not suitable for using in small clinics for children. The complexity of the sample collection method negatively affects the usability of IC. Self-blow nasal discharge specimens are relatively easy to collect by the plastic wrap, especially from children. However, viruses from nasal discharge samples are relatively difficult to detect using colloidal gold IC methods such as Prorast. FLIC-AB functions as a powerful tool for samples such as self-blow nasal discharge specimens. FLIC-AB also has an advantage of LOD over colloidal gold ICs for diagnosis in the early stage of onset, indicating that FLIC-AB may play an important role in the early-onset detection of pandemic influenza viruses and in the effective treatment of infected patients with anti-influenza virus drugs.

Interestingly, our results indicated that sensitivities of colloidal gold IC such as Prorast (over 79.5%) are lower than those of FLIC-AB, but higher in this study than those reported previously (61.6%) [36]. We predict that the condition of suspension buffer, membrane, and antibodies were the reason why conventional IC has dramatically improved since 2006, when a report was published by Keitel *et al*. [42].

In 2013, WHO defined four new phases (Inter-pandemic phase, Alert phase, Pandemic phase and Transition phase) of pandemic disease [43]. The Alert phase is defined as the time when influenza caused by a new subtype has been identified in humans. At the Alert phase, rapid identification of the new strain in humans and poultry is critical for regulatory preparedness. The FLIC method can play an important role in diagnosing infected patients and poultry at the Alert stage because this method, is more sensitive than standard IC methods, is rapid, and is easy-to-use, and function as a powerful tool for containment of pandemic viruses. At the Pandemic phase, infected patients should be identified immediately because early treatment of high-risk patients with antiviral drugs was reported to be beneficial [44]. The sensitivity and rapidity of FLICs are critical for the timely decision to use anti-viral drug treatment at the onset of disease. Taken together, FLIC technology could play an important role in influenza diagnosis at both the Alert phase and the Pandemic phase.

## Materials and Methods

### Cells and virus strains

Madin–Darby canine kidney cells (MDCK cells; American Type Culture Collection, ATCC, VA, USA) were maintained in Dulbecco’s modified Eagle’s medium (D-MEM) supplemented with 10% fetal calf serum (FCS) and penicillin-streptomycin solution.

All influenza virus strains used in this study are listed in Tables 1, 2, and 3. These viruses were described previously [37,39]. All viruses were grown in MDCK cells or 10-day-old embryonated chicken eggs.

### Development of FLIC-AB

Immobilized mouse monoclonal anti-IAV NP clone 56–1 (subclass IgG2a) and conjugated clone A43–6 (subclass IgG2a) were used for detection of IAVs. Immobilized mouse monoclonal anti-IBV NP clone 47–23 (subclass IgG2b) and conjugated clone B58–17 (subclass IgG2a) were used for detection of IBVs. Anti-NP for influenza A or B viruses was conjugated with fluorescent latex beads (3602–613, Fujikura Kasei Co., LTD, Tokyo, Japan). The conjugation protocols were described previously [37].

### Ethics Statement and Collection of samples from patients with flu symptoms

Clinical research was conducted according to the Declaration of Helsinki Principles. Protocols for sample collection, storage, and IC-based detection of influenza A or B viruses in samples obtained from patients were approved by the Institutional Review Boards (IRB approval number: 21–1 on March 25, 2011 by Ethical Committee, Tokyo Metropolitan Institute of Medical Science) at each hospital and institute, located at Tokyo, Hokkaido, Oita and other prefectures (S5 Table). Under written informed consent, samples were collected from nasal swabs, self-blow nasal discharge specimens, and nasopharyngeal aspirates of patients who had a diagnosis of influenza-like respiratory disease on the basis of signs and symptoms, such as fever. Transportation of clinical samples to Tokyo Metropolitan Institute of Medical Science was performed according to the guidelines provided by the National Institute of Infectious Diseases (Tokyo, Japan). Sequence analysis of viral genome RNA was approved by the Research Ethical Committee at Tokyo Metropolitan Institute of Medical Science (Approved Number: 12–034. Date: March 30, 2012).

### Sample preparation and viral isolation from specimens

Samples for IC assays were suspended in 500 μL of IC dilution buffer (50 mM Tris-HCl (pH 7.2), 150 mM NaCl, 1% Triton X-100), and dropped on the sample pad of each IC strip. Fluorescence was detected with a Konica Minolta immunochromatography reader (Konica Minolta, Inc., Tokyo, Japan) [37].

For qRT-PCR measurement, samples were suspended in 140 μL of D-MEM or phosphate-buffered saline (PBS). Total RNA was isolated from the suspended solution using QIAamp viral RNA Mini Kit (Qiagen, Hilden, Germany; http://www.qiagen.com/). qRT-PCR was conducted using a CFX96 Real-Time PCR Detection System (Promega, Madison, WI, USA; http://www.promega.com) and protocols described previously [39]. AmpliRun INFLUENZA A H1 RNA CONTROL (Vircell Microbiologists, Granada, Spain, http://en.vircell.com/; A/Brisbane/59/2007(H1N1)) or AmpliRun INFLUENZA B RNA CONTROL (Vircell Microbiologists, Granada, Spain, http://en.vircell.com/; B/Brisbane/60/2008) were used as standard IAV or IBV RNAs, respectively.

For viral culture, samples were stored in shipping containers at 4˚C, and sent to SRL, Inc. (Tokyo, Japan) for isolation of influenza viruses using cell culture methods.

## Supporting Information

S1 Table List of common pathogens giving negative FLIC-AB results. For evaluating the specificity of FLIC-AB, common pathogens including 14 gram-positive bacteria, 8 gram negative bacteria, 3 mycoplasmas 1 fungus, and 10 viruses besides influenza viruses were tested
(DOCX)

S2 Table Comparison of clinical performance of FLIC-AB vs. qRT-PCR in typing influenza viruses from clinical samples. (DOCX)

S3 Table Comparison of clinical performance of FLIC-AB and Prorast in typing influenza viruses from clinical samples (DOCX)

S4 Table Comparison of clinical performance of FLIC-AB and Quick in typing influenza viruses from clinical samples. (DOCX)

S5 Table List of hospitals and institutes for clinical trials with IRB approval. (DOCX)

## References

[1] Wright F, Neumann G, Kawaoka Y (2007) Orthomyxoviruses. Fields Virology. Philadelphia: Wolters Kluwer, Lippincott Willams & Wilkins pp. 1691–1740.

[2] Webster RG (1997) Influenza virus: transmission between species and relevance to emergence of the next human pandemic. Arch. Virol. Suppl. 13: 105–113. 9413531 10.1007/978-3-7091-6534-8_11

[3] Yamashita M, Krystal M, Fitch WM, Palese P (1988) Influenza B virus evolution: co-circulating lineages and comparison of evolutionary pattern with those of influenza A and C viruses. Virology 163: 112–122. 3267218 10.1016/0042-6822(88)90238-3

[4] Kanegae Y, Sugita S, Endo A, Ishida M, Senya S, et al (1990) Evolutionary pattern of the hemagglutinin gene of influenza B viruses isolated in Japan: cocirculating lineages in the same epidemic season. J. Virol. 64: 2860–2865. 2335820 10.1128/jvi.64.6.2860-2865.1990 PMC249468

[5] Rota PA, Wallis TR, Harmon MW, Rota JS, Kendal AP, et al (1990) Cocirculation of two distinct evolutionary lineages of influenza type B virus since 1983. Virology 175: 59–68. 2309452 10.1016/0042-6822(90)90186-u

[6] Dawood FS, Jain S, Finelli L, Shaw MW, Lindstrom S, et al (2009) Emergence of a novel swine-origin influenza A (H1N1) virus in humans. N. Engl. J. Med. 360: 2605–2615. 10.1056/NEJMoa0903810 19423869

[7] World Health Organization (2010) Pandemic (H1N1) 2009—update 112. World Health Organization.

[8] World Health Organization (2010) H1N1 in post-pandemic period.

[9] Garske T, Legrand J, Donnelly CA, Ward H, Cauchemez S, et al (2009) Assessing the severity of the novel influenza A/H1N1 pandemic. B. M. J. 339: b2840 10.1136/bmj.b2840 19602714

[10] Fraser C, Donnelly CA, Cauchemez S, Hanage WP, Van Kerkhove MD, et al (2009) Pandemic potential of a strain of influenza A (H1N1): early findings. Science 324: 1557–1561. 10.1126/science.1176062 19433588 PMC3735127

[11] Presanis AM, De Angelis D, Hagy A, Reed C, Riley S, et al (2009) The severity of pandemic H1N1 influenza in the United States, from April to July 2009: a Bayesian analysis. PLoS Med. 6: e1000207 10.1371/journal.pmed.1000207 19997612 PMC2784967

[12] Tuite AR, Greer AL, Whelan M, Winter AL, Lee B, et al (2010) Estimated epidemiologic parameters and morbidity associated with pandemic H1N1 influenza. C. M. A. J. 182: 131–136. 10.1503/cmaj.091807 PMC2817319 19959592

[13] Kamigaki T, Oshitani H (2009) Epidemiological characteristics and low case fatality rate of pandemic (H1N1) 2009 in Japan. PLoS Curr. 1: RRN1139 20043033 10.1371/currents.RRN1139 PMC2797432

[14] Shu B, Garten R, Emery S, Balish A, Cooper L, et al (2012) Genetic analysis and antigenic characterization of swine origin influenza viruses isolated from humans in the United States, 1990–2010. Virology 422: 151–160. 10.1016/j.virol.2011.10.016 22078166

[15] Centers for Disease Control and Prevention (2012) Influenza A (H3N2) Variant Virus-Related Hospitalizations–Ohio, 2012. MMWR Morb. Mortal. Wkly. Rep. 61: 764–767. 23013722

[16] Bowman AS, Sreevatsan S, Killian ML, Page SL, Nelson SW, et al (2012) Molecular evidence for interspecies transmission of H3N2pM/H3N2v influenza A viruses at an Ohio agricultural fair, July 2012. Emerging Microbes and Infections 1: e33. 26038404 10.1038/emi.2012.33 PMC3630945

[17] Centers for Disease Control and Preventation (2013) First H3N2v Outbreak of 2013 Reported; CDC Continues to Urge High Risk People to Avoid Swine at Fairs

[18] Indiana State Department of Health (2013) Health Officials Encourage Hoosiers to Protect Themselves From Swine Flu.

[19] Claas EC, Osterhaus AD, van Beek R, De Jong JC, Rimmelzwaan GF, et al (1998) Human influenza A H5N1 virus related to a highly pathogenic avian influenza virus. Lancet 351: 472–477. 9482438 10.1016/S0140-6736(97)11212-0

[20] de Jong JC, Claas EC, Osterhaus AD, Webster RG, Lim WL (1997) A pandemic warning? Nature 389: 554 9335492 10.1038/39218 PMC7095477

[21] Subbarao K, Klimov A, Katz J, Regnery H, Lim W, et al (1998) Characterization of an avian influenza A (H5N1) virus isolated from a child with a fatal respiratory illness. Science 279: 393–396. 9430591 10.1126/science.279.5349.393

[22] Gambotto A, Barratt-Boyes SM, de Jong MD, Neumann G, Kawaoka Y (2008) Human infection with highly pathogenic H5N1 influenza virus. Lancet 371: 1464–1475. 10.1016/S0140-6736(08)60627-3 18440429

[23] Webster RG, Govorkova EA (2006) H5N1 influenza–continuing evolution and spread. N. Engl. J. Med. 355: 2174–2177. 17124014 10.1056/NEJMp068205

[24] World Health Organization (2013) Cumulative number of confirmed human cases for avian influenza A(H5N1) reported to WHO, 2003–2013. World Health Organization.

[25] World Health Organization (2013) Number of confirmed human cases of avian influenza A(H7N9) reported to WHO Report 9—data in WHO/HQ as of 12 August 2013, 14:45 GMT+1.

[26] Kageyama T, Fujisaki S, Takashita E, Xu H, Yamada S, et al (2013) Genetic analysis of novel avian A(H7N9) influenza viruses isolated from patients in China, February to April 2013. Euro. Surveill. 18: 20453 23594575 PMC6296756

[27] Gao R, Cao B, Hu Y, Feng Z, Wang D, et al (2013) Human infection with a novel avian-origin influenza A (H7N9) virus. N. Engl. J. Med. 368: 1888–1897. 10.1056/NEJMoa1304459 23577628

[28] Liu D, Shi W, Shi Y, Wang D, Xiao H, et al (2013) Origin and diversity of novel avian influenza A H7N9 viruses causing human infection: phylogenetic, structural, and coalescent analyses. Lancet 381: 1926–1932. 10.1016/S0140-6736(13)60938-1 23643111

[29] Chen Y, Liang W, Yang S, Wu N, Gao H, et al (2013) Human infections with the emerging avian influenza A H7N9 virus from wet market poultry: clinical analysis and characterisation of viral genome. Lancet 381: 1916–1925. 10.1016/S0140-6736(13)60903-4 23623390 PMC7134567

[30] Watanabe T, Kiso M, Fukuyama S, Nakajima N, Imai M, et al (2013) Characterization of H7N9 influenza A viruses isolated from humans. Nature 501: 551–555. 10.1038/nature12392 23842494 PMC3891892

[31] Mok CK, Lee HH, Chan MC, Sia SF, Lestra M, et al (2013) Pathogenicity of the novel A/H7N9 influenza virus in mice. MBio. 4 10.1128/mBio.01006-13 23820393 PMC3705449

[32] Belser JA, Gustin KM, Pearce MB, Maines TR, Zeng H, et al (2013) Pathogenesis and transmission of avian influenza A (H7N9) virus in ferrets and mice. Nature 501: 556–559. 10.1038/nature12391 23842497 PMC7094885

[33] Sakurai A, Shibasaki F (2012) Updated values for molecular diagnosis for highly pathogenic avian influenza virus. Viruses 4: 1235–1257. 23012622 10.3390/v4081235 PMC3446759

[34] Sakai-Tagawa Y, Ozawa M, Tamura D, Le MQ, Nidom CA, et al (2010) Sensitivity of influenza rapid diagnostic tests to H5N1 and 2009 pandemic H1N1 viruses. J. Clin. Microbiol. 48: 2872–2877. 10.1128/JCM.00439-10 20554831 PMC2916590

[35] Sasaki T, Kubota-Koketsu R, Takei M, Hagihara T, Iwamoto S, et al (2012) Reliability of a newly-developed immunochromatography diagnostic kit for pandemic influenza A/H1N1pdm virus: implications for drug administration. PLoS One 7: e50670 10.1371/journal.pone.0050670 23226350 PMC3511324

[36] Cruz AT, Cazacu AC, McBride LJ, Greer JM, Demmler GJ (2006) Performance characteristics of a rapid immunochromatographic assay for detection of influenza virus in children during the 2003 to 2004 influenza season. Ann. Emerg. Med. 47: 250–254. 16492491 10.1016/j.annemergmed.2005.10.019

[37] Sakurai A, Takayama K, Nomura N, Munakata T, Yamamoto N, et al (2013) Broad-spectrum detection of h5 subtype influenza a viruses with a new fluorescent immunochromatography system. PLoS One 8: e76753 10.1371/journal.pone.0076753 24223117 PMC3819354

[38] Shichinohe S, Okamatsu M, Yamamoto N, Noda Y, Nomoto Y, et al (2013) Potency of an inactivated influenza vaccine prepared from a non-pathogenic H5N1 virus against a challenge with antigenically drifted highly pathogenic avian influenza viruses in chickens. Vet. Microbiol. 164: 39–45. 10.1016/j.vetmic.2013.01.041 23462521

[39] Sakurai A, Nomura N, Nanba R, Sinkai T, Iwaki T, et al (2011) Rapid typing of influenza viruses using super high-speed quantitative real-time PCR. J. Virol. Methods 178: 75–81. 10.1016/j.jviromet.2011.08.015 21889540 PMC7119501

[40] Chan KH, Lam SY, Puthavathana P, Nguyen TD, Long HT, et al (2007) Comparative analytical sensitivities of six rapid influenza A antigen detection test kits for detection of influenza A subtypes H1N1, H3N2 and H5N1. J. Clin. Virol. 38: 169–171. 17194622 10.1016/j.jcv.2006.11.010

[41] Wada A, Sakoda Y, Oyamada T, Kida H (2011) Development of a highly sensitive immunochromatographic detection kit for H5 influenza virus hemagglutinin using silver amplification. J. Virol. Methods 178: 82–86. 10.1016/j.jviromet.2011.08.017 21911008

[42] Keitel K, Wagner N, Lacroix L, Manzano S, Gervaix A (2011) Performance characteristics of a rapid immunochromatographic assay for detection of pandemic influenza A (H1N1) virus in children. Eur. J. Pediatr. 170: 511–517. 10.1007/s00431-010-1326-0 20938682

[43] World Health Organization (2013) Pandemic Influenza Risk Management WHO Interim Guidance. WHO.

[44] Jain S, Kamimoto L, Bramley AM, Schmitz AM, Benoit SR, et al (2009) Hospitalized patients with 2009 H1N1 influenza in the United States, April-June 2009. N. Engl. J. Med. 361: 1935–1944. 10.1056/NEJMoa0906695 19815859

