# Supplementary material for: Fluorescent Immunochromatography for Rapid and Sensitive Typing of Seasonal Influenza Viruses
Source: PLoS One. 2015 Feb 4;10(2):e0116715. doi: 10.1371/journal.pone.0116715 (PMC4317186; doi:10.1371/journal.pone.0116715)
Supplement: S1 Table — For evaluating the specificity of FLIC-AB, common pathogens including 14 gram-positive bacteria, 8 gram negative bacteria, 3 mycoplasmas 1 fungus, and 10 viruses besides influenza viruses were tested (DOCX) [file pone.0116715.s001.docx]

**Table S1:** List of common pathogens giving negative FLIC-AB results

| **Tested bacteria** |
| --- |
| **Gram-positive bacteria** |
| *Corynebacterium diphtheriae* |
| *Enterococcus faecalis* |
| *Listeria monocytogenes* |
| *Staphylococcus aureus* |
| *Staphylococcus epidermidis* |
| *Streptococcus agalactiae* (Group B) |
| *Streptococcus anginosus* (Group F) |
| *Streptococcus dysgalactiae subsp. equisimilis* (Group C) |
| *Streptococcus dysgalactiae subsp. equisimilis* (Group G) |
| *Streptococcus mutans* |
| *Streptococcus pneumonia* |
| *Streptococcus pyogenes* (Group A) |
| *Streptococcus sanguis* |
| *Mycoplasma hominis* |
| **Gram-negative bacteria** |
| *Bordetella pertussis* |
| *Escherichia coli* |
| *Haemophilus influenzae* |
| *Klebsiella pneumoniae* |
| *Moraxella cartarrhalis* |
| *Proteus vulgaris* |
| *Pseudomonas aeruginosa* |
| *Serratia marcescens* |
| **Mycoplasma** |
| *Mycoplasma hominis* |
| *Mycoplasma pneumonia* (strain FH) |
| *Mycoplasma salivarium* |
| **Tested Fungi** |
| *Candida albicans* |
| **Tested Viruses** |
| Adenovirus Type3 (strain AD3) |
| Coxsackie virus Type A2 (strain 5-348-SMCA2) |
| Coxsackie virus Type A3 (strain 7-339-RCA3) |
| Coxsackie virus Type A4 (strain 7-435-SMCA4) |
| Coxsackie virus Type B4 (strain 8-184-L-CB4) |
| Coxsackie virus Type B5 (strain 8-32-CB5) |
| Echovirus Type 4 (strain 8-15T-R-E4) |
| Echovirus Type 7 (strain 8-93-LR-E7) |
| Echovirus Type 16 (strain 7-540-R-E16) |
| Respiratory syncytial virus (strain subgroup A/long) |
