# Supplementary material for: Fluorescent Immunochromatography for Rapid and Sensitive Typing of Seasonal Influenza Viruses
Source: PLoS One. 2015 Feb 4;10(2):e0116715. doi: 10.1371/journal.pone.0116715 (PMC4317186; doi:10.1371/journal.pone.0116715)
Supplement: S2 Table — (DOCX) [file pone.0116715.s002.docx]

**Table S2.** Comparison of clinical performance of FLIC-AB vs. qRT-PCR in typing influenza viruses from clinical samples

**Nasal swab samples**

|  | | **Results of qRT-PCR** | |  |
| --- | --- | --- | --- | --- |
| **Results of FLIC-AB** | | Positive | Negative | Total |
| **Influenza A** | Positive | 74 | 0 | 74 |
|  | Negative | 1 | 54 | 55 |
|  | Total | 75 | 54 | 129 |
| **Influenza B** | Positive | 90 | 1 | 91 |
|  | Negative | 0 | 54 | 54 |
|  | Total | 90 | 55 | 145 |

**Self-blow nasal discharge specimens**

|  | | **Results of qRT-PCR** | |  |
| --- | --- | --- | --- | --- |
| **Results of FLIC-AB** | | Positive | Negative | Total |
| **Influenza A** | Positive | 70 | 0 | 70 |
|  | Negative | 4 | 51 | 55 |
|  | Total | 74 | 51 | 125 |
| **Influenza B** | Positive | 64 | 0 | 64 |
|  | Negative | 6 | 51 | 57 |
|  | Total | 70 | 51 | 121 |

**Nasopharyngeal aspirates**

|  | | **Results of qRT-PCR** | |  |
| --- | --- | --- | --- | --- |
| **Results of FLIC-AB** | | Positive | Negative | Total |
| **Influenza A** | Positive | 73 | 2 | 75 |
|  | Negative | 1 | 66 | 67 |
|  | Total | 74 | 68 | 142 |
| **Influenza B** | Positive | 56 | 0 | 56 |
|  | Negative | 2 | 66 | 68 |
|  | Total | 58 | 66 | 124 |
