# Supplementary material for: Fluorescent Immunochromatography for Rapid and Sensitive Typing of Seasonal Influenza Viruses
Source: PLoS One. 2015 Feb 4;10(2):e0116715. doi: 10.1371/journal.pone.0116715 (PMC4317186; doi:10.1371/journal.pone.0116715)
Supplement: S4 Table — (DOCX) [file pone.0116715.s004.docx]

**Table S4.** Comparison of clinical performance of FLIC-AB and Quick in typing influenza viruses from clinical samples

**Nasal swab samples**

|  | | **Results of Quick** | |  |
| --- | --- | --- | --- | --- |
| **Results of FLIC-AB** | | Positive | Negative | Total |
| **Influenza A** | Positive | 74 | 0 | 74 |
|  | Negative | 0 | 55 | 55 |
|  | Total | 74 | 55 | 129 |
| **Influenza B** | Positive | 94 | 5 | 99 |
|  | Negative | 0 | 52 | 52 |
|  | Total | 94 | 57 | 151 |

**Self-blow nasal discharge specimens**

|  | | **Results of Quick** | |  |
| --- | --- | --- | --- | --- |
| **Results of FLIC-AB** | | Positive | Negative | Total |
| **Influenza A** | Positive | 63 | 7 | 70 |
|  | Negative | 0 | 61 | 61 |
|  | Total | 63 | 68 | 131 |
| **Influenza B** | Positive | 60 | 4 | 64 |
|  | Negative | 0 | 61 | 61 |
|  | Total | 60 | 65 | 125 |

**Nasopharyngeal aspirates**

|  | | **Results of Quick** | |  |
| --- | --- | --- | --- | --- |
| **Results of FLIC-AB** | | Positive | Negative | Total |
| **Influenza A** | Positive | 73 | 2 | 75 |
|  | Negative | 0 | 69 | 69 |
|  | Total | 73 | 71 | 144 |
| **Influenza B** | Positive | 55 | 1 | 56 |
|  | Negative | 0 | 69 | 69 |
|  | Total | 55 | 70 | 125 |
