# Supplementary material for: Fluorescent Immunochromatography for Rapid and Sensitive Typing of Seasonal Influenza Viruses
Source: PLoS One. 2015 Feb 4;10(2):e0116715. doi: 10.1371/journal.pone.0116715 (PMC4317186; doi:10.1371/journal.pone.0116715)
Supplement: S5 Table — (DOCX) [file pone.0116715.s005.docx]

**Table S5:** List of Hospitals and Institutes for clinical trials with IRB approval

| **No.** | **Hospitals and Institutes** | **Date of Approval** | **Approval number** |
| --- | --- | --- | --- |
|  |  |  |  |
| 1 | Matsumoto Children's Clinic | 2012.4.3 | 12001 |
| 2 | Oita Children's Hospital | 2012.4.5 | 12002 |
| 3 | Kumanomido Children's Clinic | 2013.1.10 | 13001 |
| 4 | Nozaki Clinic | 2013.3.15 | 13002 |
| 5 | Uda Clinic | 2013.4.10 | 13003 |
| 6 | Ohoya Children's Clinic | 2013.4.10 | 13004 |
| 7 | Dr. Jun General Hospital for Children | 2013.4.12 | 13005 |
| 8 | Kumagae Clinic | 2013.4.15 | 13006 |
| 9 | Yanagi Clinic | 2013.4.15 | 13007 |
| 10 | Hukuda Children's Clinic | 2013.4.15 | 13008 |
| 11 | Takada Central Hospital | 2013.4.18 | 13009 |
| 12 | Michino Clinic | 2013.4.18 | 13010 |
| 13 | Inoue Clinic | 2013.4.19 | 13011 |
| 14 | Matsui Children's Clinic | 2013.4.26 | 13012 |
| 15 | Koizumi Clinic for Respiratory and internal Medicine | 2013.5.12 | 13001 |
| 16 | Ibaraki Prefectural Central Hospital | 2012.12.19 | 12001 |
| 17 | Moriya Keiyu Hospital | 2013.2.21 | 13001 |
| 18 | Koshigaya City Hospital | 2013.1.18 | 13001 |
| 19 | Hitachinaka General Hospital | 2012.12.19 | 12001 |
| 20 | Meiwa Hospital | 2012.12.19 | 12001 |
| 21 | Kenko-mori Clinic | 2012.12.19 | 12001 |
| 22 | Arakawa Children's Clinic | 2013.4.19 | 13001 |
| 23 | Tokyo Metropolitan Komagome Hospital | 2010.3.25 | 818 |
| 24 | Tokyo Metropolitan institute of Medical Science | 2011.3.25 | 21-1 |
